# Distinct substrate specificities of *Arabidopsis* DCL3 and DCL4

**DOI:** 10.1093/nar/gkt1077

**Published:** 2013-11-07

**Authors:** Hideaki Nagano, Akihito Fukudome, Akihiro Hiraguri, Hiromitsu Moriyama, Toshiyuki Fukuhara

**Affiliations:** Department of Applied Biological Sciences, Tokyo University of Agriculture and Technology, 3-5-8 Saiwaicho, Fuchu, Tokyo 183-8509, Japan

## Abstract

In *Arabidopsis thaliana*, Dicer-like 3 (DCL3) and Dicer-like 4 (DCL4) cleave long, perfect double-stranded RNAs (dsRNAs) into 24 and 21 nucleotides (nt) small interfering RNAs, respectively, which in turn function in RNA-directed DNA methylation and RNA interference, respectively. To reveal how DCL3 and DCL4 individually recognize long perfect dsRNAs as substrates, we biochemically characterized DCL3 and DCL4 and compared their enzymatic properties. DCL3 preferentially cleaves short dsRNAs with 5′ phosphorylated adenosine or uridine and a 1 nt 3′ overhang, whereas DCL4 cleaves long dsRNAs with blunt ends or with a 1 or 2 nt 3′ overhang with similar efficiency. DCL3 produces 24 nt RNA duplexes with 2 nt 3′ overhangs by the 5′ counting rule. Inorganic phosphate, NaCl and KCl enhance DCL3 activity but inhibit DCL4 activity. These results indicate that plants use DCLs with distinct catalytic profiles to ensure each dsRNA substrate generates only a specific length of siRNAs that trigger a unique siRNA-mediated response.

## INTRODUCTION

A hallmark of RNA silencing is production of small RNAs 20–30 nucleotides (nt) long that are processed from long double-stranded RNA (dsRNA) precursors by Dicer proteins (1–4). Studies on Dicer activities and functions are one of the most intensive subjects for elucidating the molecular mechanisms of RNA silencing. Dicers from several model organisms have been biochemically characterized (5–9). These Dicers cleave pre-miRNAs containing stem-loop structures or long perfect dsRNAs into microRNA (miRNA) duplexes or small interfering RNA (siRNA) duplexes of 20–25 nt, which possess 5′ monophosphates, 3′ hydroxyl groups and 2 nt 3′ overhangs (10–13). Human Dicer produces both miRNAs and siRNAs (6), whereas two *Drosophila* Dicers, Dicer-1 and Dicer-2, respectively, produce miRNAs and siRNAs (14). Substrate recognition mechanisms of human Dicer and the two *Drosophila* Dicers have been extensively studied biochemically (15–17).

Animals and fungi have only one or two Dicer proteins, but plants, such as the model plant *Arabidopsis thaliana*, have at least four distinct Dicers (Dicer-like 1 through 4, DCL1–4) (18). Functions of plant DCLs have mainly been revealed from genetic studies using mutant *Arabidopsis* plants (19,20). DCL1 produces miRNAs (19), and DCL2–DCL4 produce siRNAs with specific sizes, respectively, 22, 24 and 21 nt, from long perfect dsRNAs (20). These three siRNA-generating DCLs function distinctly but in part redundantly in each RNA silencing pathway *in vivo* (21). For instance, DCL2, DCL3 and DCL4, respectively, produce natural antisense siRNAs (nat-siRNAs), heterochromatic siRNAs (hc-siRNAs) and trans-acting siRNAs (ta-siRNAs), but DCL4, DCL2 and also DCL3, redundantly produce viral siRNAs to defend against virus infection (22–25).

Both DCL3 and DCL4 have important functions *in vivo*, because their products, 24 and 21 nt siRNAs, function distinctly (20,26). DCL3 products function in transcriptional gene silencing (TGS) via RNA-directed DNA methylation (RdDM) to suppress activation of transposons, whereas DCL4 products function in post-transcriptional gene silencing (PTGS or RNA interference) via sequence-specific mRNA cleavage or translational inhibition to regulate gene expression and defend against viral infections. Indeed, in animals, Piwi-interacting RNAs (piRNAs), which are a different class of small RNAs produced by a Dicer-independent pathway, function to suppress transposon activation, whereas siRNAs produced by Dicer defend against viral infections (27). Therefore, plant 24 nt siRNAs produced by DCL3 are functionally equivalent of animal piRNAs.

Although DNA methylation and mRNA cleavage are individually crucial and completely different reactions, in plants each reaction is induced by similar siRNAs with specific lengths (24 and 21 nt) that are produced from similar precursor dsRNAs by DCL3 and DCL4, respectively. Therefore, DCL3 and DCL4 must individually recognize proper substrate dsRNAs generated from genomic regions with inserted transposable elements and viral genomes, respectively, to produce 24 and 21 nt siRNAs. Therefore, revealing the mechanisms of substrate recognition by DCL3 and DCL4 of long perfect dsRNAs is important for understanding their proper sharing of important roles *in vivo*. However, substrate recognition mechanisms of plant siRNA-generating Dicers (DCL2-4) have never been reported, because biochemical characterization of DCLs is limited (28–30).

We have simultaneously detected DCL3 and DCL4 activities in crude extracts from seedlings of *A. thaliana* (31). Because DCL4 activity is detected at much higher levels than DCL3 activity, recently we biochemically characterized the dsRNA-cleaving activity of DCL4 using long perfect dsRNAs of ∼500 nt as substrates (31). In this study, we prepared various short dsRNAs of 30–55 nt long as substrates for Dicer assays. To reveal how DCL3 and DCL4 individually recognize perfect dsRNAs as substrates and, respectively, produce 24 and 21 nt siRNAs, we biochemically characterized DCL3 and DCL4 and compared their enzymatic properties. Our results demonstrated that the substrate specificities of DCL3 and DCL4 differ distinctly, just as their *in vivo* functions differ.

## MATERIALS AND METHODS

### Plant materials and growth conditions

*Arabidopsis thaliana* plants were grown on solid MS medium in a controlled-environment chamber under the following conditions: 40–50 μmol photons m^−^^2 ^sec^−^^1^ irradiance, 16 h light and 8 h dark, at 22°C after 3 days at 4°C. *dcl3-1* (SALK_005512) and *dcl4-2* (GABI 160G05) were provided by the Salk T-DNA collection and the Genomanalyse im Biologischen System Pflanze, respectively.

### dsRNA preparation

Single-stranded RNAs (ssRNAs) <55 nt long were synthesized by Japan Bio Services Co. (Saitama, Japan), and end-labeled by T4 polynucleotide kinase (Takara, Japan) and [γ-^32^P]-ATP. Equal amounts of ^32^P-labeled sense RNA (0.01 pmol) and antisense RNA were annealed in 10 mM Tris-HCl (pH 7.5) and 100 mM NaCl by heating at 90°C for 5 min, followed by turning off the heater for 10 min and incubating at room temperature for 10 min. dsRNAs were purified by phenol/chloroform, precipitated in ethanol, and dissolved in sterilized water prior to use.

Preparation of [α-^32^P]-UTP-labeled long dsRNAs of 100 or 500 nt with blunt ends was described previously (31). Briefly, [α-^32^P]-UTP-labeled sense and antisense transcripts were synthesized using RiboMAXTM T7 Large Scale RNA Production Systems (Promega), and then equal amounts of ^32^P-labeled RNAs were annealed. Template DNA and ssRNA were digested by S1 nuclease (TaKaRa) and DNase I (Promega).

### Dicer (dsRNA cleaving) assay

Two-week-old *Arabidopsis* seedlings were collected and homogenized in 3 ml g^−^^1^ of extraction buffer containing 20 mM Tris-HCl (pH 7.5), 4 mM MgCl_2_, 5 mM DTT, 1 mM phenylmethylsulfonyl fluoride, 1 μg ml^−^^1^ leupeptin and 1 μg ml^−^^1^ pepstatin A at 4°C. Homogenates were centrifuged twice at 20000*g* at 4°C for 10–15 min to remove debris, and the supernatant was collected as crude extract (31). ^32^P-labeled dsRNAs (final concentration ∼0.5 nM) were incubated with 15 μl of crude extract at 22°C for 2 h in 20 μl of dsRNA-cleaving buffer containing 30 mM Tris-HCl (pH 7.5), 50 mM NaCl, 4 mM MgCl_2_, 5 mM ATP and 1 mM GTP. Additionally, 0.5 μl of RNaseOUT (Invitrogen) was added to each 20-μl reaction. After incubation, the cleavage products were purified by phenol/chloroform, separated by 15% denaturing PAGE with 8 M urea, and detected by autoradiography.

Small RNA products were quantitated from relative band intensities measured with a Typhoon FLA 7000 image analyzer (GE Healthcare). The 21 nt RNA producing activity (DCL4 activity) was calculated as the relative band intensity of 21 nt RNA with respect to the intensity of all bands including smeary bands in each lane, and the 24 nt RNA producing activity (DCL3 activity) was calculated using the same equation.

## RESULTS

### DCL3 and DCL4 preferentially cleave short and long dsRNAs, respectively

**Figure 1.**
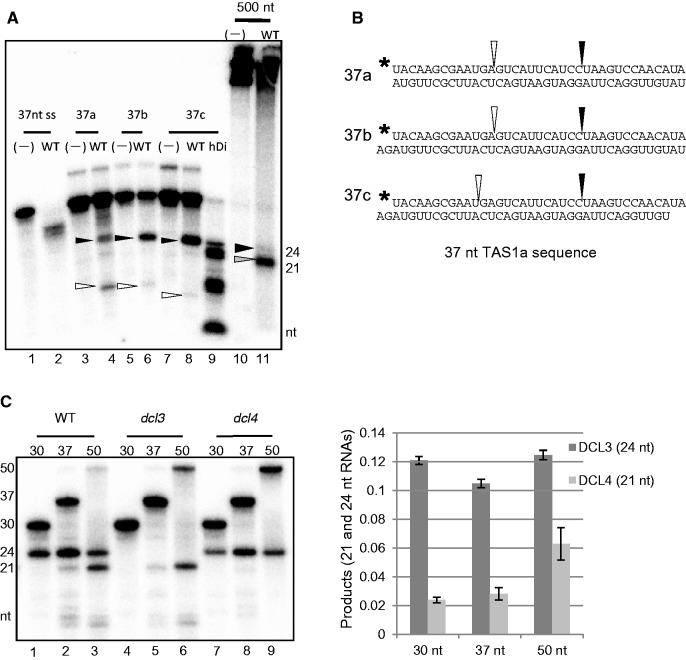
DCL3 and DCL4 preferentially cleave short and long dsRNAs, respectively. (**A**) DCL3 efficiently cleaves short dsRNAs 37 nt long. For a Dicer assay, ^32^P-labeled dsRNAs of 37 and 500 nt were incubated with crude extracts from WT *Arabidopsis* seedlings for 2 h at 22°C. Cleavage products were analysed by 15% denaturing PAGE. *Abbreviations*: 37 nt ss, 37 nt single-stranded RNA; 37 a, 37 b and 37 c, 37 nt dsRNAs with various ends indicated in (**B**). 37 nt ssRNA and dsRNAs were phosphorylated at the 5′ end by [γ-^32^P]-ATP. 500 nt, 500 nt dsRNA whole-labeled by ^32^P-UTP. (–), no crude extracts (negative control). A recombinant human Dicer fragment (hDi) was used as a cleavage control to generate size markers. Black (24 nt) and white (11 or 13 nt) arrowheads indicate cleavage products produced by DCL3, and a gray arrowhead (21 nt) indicates cleavage products by DCL4. (B) Structures of substrate dsRNAs of 37 nt, which have a *TAS1a* gene sequence, and cleavage sites (black and white arrowheads) by DCL3. Black stars indicate ^32^P. (**C**) DCL3 cleaves short dsRNAs, whereas DCL4 cleaves long dsRNAs. 5′-^32^P-phosphorylated dsRNAs of 30, 37 and 50 nt with 2 nt 3′ overhangs were incubated with crude extracts from WT, *dcl3-1* or *dcl4-2 Arabidopsis* seedlings for 2 h at 22°C. Autoradiography (left) results are shown. The 21 nt RNA producing activity (DCL4 activity) was calculated as the relative band intensity of 21 nt RNA with respect to the intensity of all bands including smeary bands in each lane, and the 24 nt RNA producing activity (DCL3 activity) was calculated using the same equation. Values are means ± standard deviation for three independent experiments (right).

We carried out Dicer assays with 37 and 500 nt dsRNAs as substrates on crude extracts isolated from *Arabidopsis* seedlings. To mimic different processing intermediates, three different forms of 37 nt dsRNA with distinct terminal overhangs were prepared, i.e., two blunt ends (37 a), one 2 nt 3′ overhang and one blunt end (37 b), and two 2 nt 3′ overhangs (37 c) (Figure 1B). Cleavage products were analysed by denaturing PAGE (Figure 1A). Incubation of any of 37 nt dsRNAs with wild type extract (WT) produced 24 nt cleavage products with only trace amount of 21 nt products (black arrowheads on lanes 4, 6 and 8 in Figure 1A). The highest dicing activity was observed with 37 c substrate containing two 2 nt 3′ overhangs. In contrast, when 500 nt dsRNA was used as a substrate, a predominant 21 nt cleavage products (gray arrowhead on lane 11 in Figure 1A), but only trace amount of 24 nt product (black arrowhead on lane 11 in Figure 1A) were detected. Incubation of 37 nt single-stranded RNA with WT extract produced ∼30 nt cleavage products (lane 2 in Figure 1A) but not specific dicing products, indicative of digestion by an unknown ribonuclease activity.

We have previously established that the 24 and 21 nt fragments generated from 500 nt dsRNA by WT extracts are products of endogenous DCL3 and DCL4, respectively (31). To analyse isoform-specific dicing activity for short dsRNAs, dicing activities of extracts prepared from WT, *dcl3-1* or *dcl4-2* plants and immunoprecipitated DCL3 and DCL4 were analysed. Using 5′-^32^P-phosphorylated dsRNAs of 30, 37 and 50 nt with 2 nt 3′ overhangs as substrates, we observed that WT extract could produce the 24 nt fragment from all three substrates whereas production of the 21 nt fragment was detectable only with 37 nt, albeit weakly and 50 nt dsRNA substrates (Figure 1C). Consequently, cleavage products from 30 or 37 nt dsRNA were mainly 24 nt (lanes 1 and 2), but cleavage products from 50 nt dsRNA were a mixture of 21 and 24 nt (lane 3). Therefore, a 50 nt substrate was degraded more than 30 and 37 nt substrates, because it was cleaved by both DCLs. With *dcl3* extract (lanes 4–6), no 24 nt cleavage products were detected, and with *dcl4* extract (lanes 7–9), no 21 nt cleavage products were detected (Figure 1C). Consistently, immunecomplexes precipitated with an anti-DCL3 antibody effectively produced the 24 nt product from a 37 nt substrate but less so from 100 or 500 nt substrates, whereas that precipitated with an anti-DCL4 antibody specifically produced the 21 nt product (Supplementary Figure S1C). These results indicate that DCL3 preferentially cleaves shorter dsRNAs (30–50 nt) into 24 nt RNA but DCL4 preferentially cleaves longer dsRNAs (50–500 nt) into 21 nt RNA (Supplementary Figure S1D). Although DCL1 is known to generate 21 nt dsRNAs (30), the 21 nt fragment was only detected in the presence of DCL4, and DCL1 activity was not detectable in this assay.

Substrate specificity of DCL3 dicing activity was further examined by comparing the reaction products generated from three forms of 37 nt substrates (Figure 1A). With blunt-ended dsRNA substrate (37 a) similar levels of 24 and 13 nt P^32^-labeled bands were observed, indicating DCL3 accessed 5′-phosphorylated and 5'-non-phosphorylated ends with similar efficacy. With substrates containing a 5′-phosphorylated end with 2 nt 3′ overhang (37 b and 37 c; Figure 1B), a greater amount of 24 nt cleavage products (indicated by black arrowheads) was produced than 11 or 13 nt cleavage products (indicated by white arrowheads), indicating that DCL3 preferentially accessed a 5′-phosphorylated end regardless of a 3′-overhang or blunt terminus at a 5'-non-phosphorylated end. These results establish that DCL3 preferentially accesses 5′-phosphorylated ends with 2 nt 3′ overhangs, but its recognition of 5′-phosphorylated nucleotides on blunt-ended dsRNAs is not strict.

### DCL3 but not DCL4 preferentially cleaves short dsRNAs with 5′ phosphorylated adenosine or uridine

The above results indicated that DCL3 specifically recognize a 5′-phosphorylation and 3'-overhang structure of dsRNA substrate. Since these structures are potentially important determinants of accurate production of siRNAs (16), we characterized substrate structural requirements at the 5' phosphorylated end for efficient recognition by DCL3.

**Figure 2.**
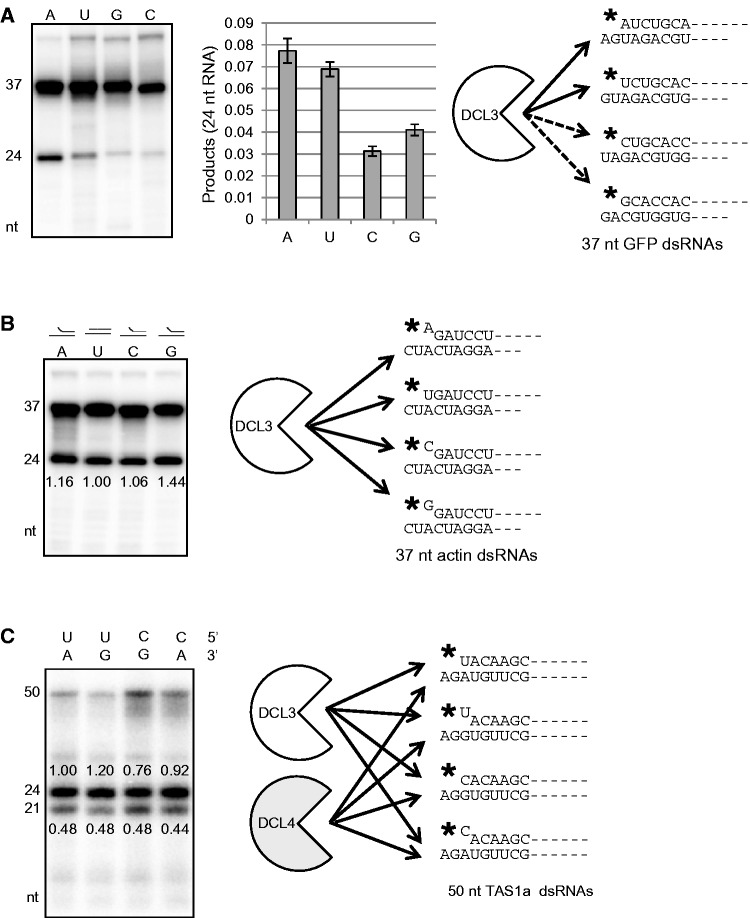
DCL3 preferentially cleaves 37 nt dsRNAs with adenosine or uridine at their 5′ phosphorylated ends. (**A**) DCL3 cleaves short dsRNAs with adenosine or uridine at their 5′ phosphorylated ends. Four 37 nt dsRNAs, which have a GFP gene sequence containing four different nucleotides at the 5′ phosphorylated end and 2 nt 3′ overhang, were used as substrates for Dicer assays. Cleavage products were analysed by 15% denaturing PAGE. The 24 nt RNA producing activity (DCL3 activity) was calculated as the relative band intensity of 24 nt RNA with respect to the intensity of all bands in each lane following autoradiography, and values are means ± standard deviation for three independent experiments. The schematic figure shows cleavage preference of DCL3. Solid arrows indicate cleavage preference of DCL3. Black stars indicate ^32^P. (**B**) DCL3 efficiently cleaves short dsRNAs with an unstable end. Three 37 nt dsRNAs containing a mismatched base pair at their 5′ phosphorylated ends and one 37 nt perfect dsRNA containing uridine (U) at the 5′ phosphorylated end were used as substrates for Dicer assays. Amounts of cleavage products (24 nt RNA) were calculated as the relative band intensity of 24 nt RNA with respect to the intensity of all bands in each lane following autoradiography. Values in each lane are relative signal intensities of cleavage products, defining the signal intensity in lane U as 1.00. (**C**) No cleavage preference of DCL3 or DCL4 for 50 nt dsRNAs with various ends as substrates. Four 50 nt dsRNAs with various base pairs at 5′ ^32^P-labeled ends (see right figure) and 2 nt 3′ overhangs were used as substrates for Dicer assays. Amounts of cleavage products, 21 nt RNA (DCL4) and 24 nt RNA (DCL3), were calculated as the relative band intensities of 21 and 24 nt RNAs, respectively, with respect to the intensity of all bands in each lane following autoradiography. Values in each lane are relative signal intensities of cleavage products, defining the signal intensity of 24 nt product in lane UA as 1.00.

To examine cleavage preference of DCL3 for nucleotides at the 5′ phosphorylated end, four kinds of 37 nt dsRNAs, which have a GFP sequence containing four different nucleotides at the 5′ phosphorylated ends, were used as substrates for Dicer assays. DCL3 preferentially cleaved the dsRNAs containing adenosine (A) or uridine (U) at the 5′ phosphorylated end (Figure 2A). Similar results were also obtained from related experiments with four dsRNAs containing an actin sequence (Supplementary Figure S2). Therefore, using either dsRNAs derived from a GFP or an actin gene, DCL3 preferentially cleaved the dsRNAs containing adenosine (A) or uridine (U) at the 5′ phosphorylated end (Figure 2A and Supplementary Figure S2).

Since an A:U base pair is less stable than a G:C base pair, we anticipated that DCL3 may tend to access less stable termini of dsRNAs. To examine this possibility, 37 nt dsRNAs containing a mismatched base pair at the phosphorylated end were used as substrates for Dicer assays. DCL3 cleaved dsRNAs containing a mismatched base pair as well as an A:U base pair, even though they contained guanosine (G) or cytosine (C) at the 5′ phosphorylated end (Figure 2B), suggesting that DCL3 can recognize the stability of the phosphorylated end of dsRNAs and preferentially accesses less stable ends.

To examine substrate recognition by DCL4, 50 nt dsRNAs with various 5′ structures were used as substrates for Dicer assays. DCL4 cleaved four kinds of dsRNAs, which contained U:A, U:G, C:G or C:U (mismatched) base pairs at the 5′ phosphorylated end, with similar efficiency (21 nt signals in Figure 2C), suggesting that the base pair or base species at the 5′ phosphorylated end does not affect DCL4 activity.

The Dicer assay using 50 nt dsRNA substrate could detect DCL3 activity as well. Surprisingly, the base pair or base species at the 5′ phosphorylated end did not affect DCL3 activity toward 50 nt dsRNAs either (24 nt signals in Figure 2C). Therefore, DCL3 activity toward 37 nt dsRNAs was affected by the terminal base pairs (Figure 2A and B) but its activity toward 50 nt dsRNAs was not affected by the terminal base pairs (Figure 2C). Stability of the terminal base pair in substrate dsRNAs may affect DCL3 activity only when the length of substrate dsRNAs is <37 nt. Because the double-stranded structure of 50 nt dsRNA is more stable than that of 37 nt dsRNA, stability of the terminal base pair in longer stable dsRNAs may not affect DCL3 activity.

### DCL3 preferentially cleaves dsRNAs with a 1 nt 3′ overhang

**Figure 3.**
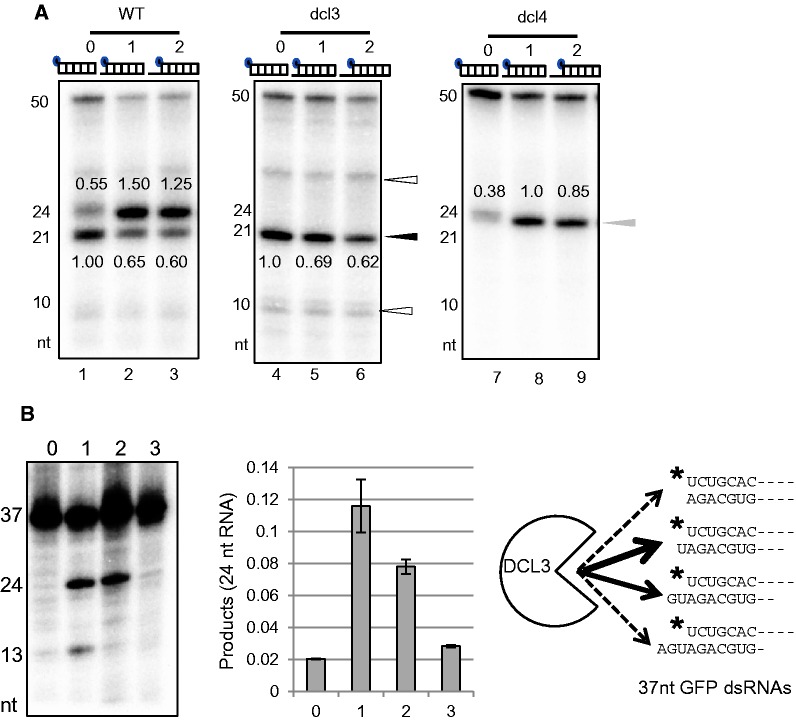
DCL3 cleaves dsRNAs with 1 nt 3′ overhang more efficiently than dsRNAs with 2 nt 3′ overhang or a blunt end, but DCL4 cleaves these dsRNAs with similar efficiency. (**A**) Cleavage products of three 50 nt dsRNAs with various 3′ ends formed by DCL3 or DCL4 and analysed by denaturing PAGE. Three 50 nt dsRNAs, which had blunt ends or 1 or 2 nt 3′ overhangs and contained a *TAS1a* gene sequence, were used as substrates for Dicer assays with WT, *dcl3-1* or *dcl4-2* crude extracts. Amounts of cleavage products, 21 nt RNA (DCL4) and 24 nt RNA (DCL3), were calculated as the relative band intensities of 21 and 24 nt RNAs, respectively, with respect to the intensity of all bands in each lane following autoradiography. Values in each lane are relative signal intensities of cleavage products, defining the signal intensity of 21 or 24 nt product in lane 1, 4 or 8 as 1.00. A black arrowhead indicates 21 nt cleavage products obtained from the 5′ phosphorylated end by DCL4, and white arrowheads indicate ∼10 and 30 nt cleavage products from the non-phosphorylated end by DCL4. A gray arrowhead indicates 24 nt cleavage products from the 5′ phosphorylated end by DCL3. (**B**) DCL3 preferentially cleaves dsRNAs with a 1 nt 3′ overhang. Four 37 nt dsRNAs, which had blunt ends or 1–3 nt 3′ overhangs and contained a GFP gene sequence, were used as substrates for Dicer assays. The 24 nt RNA producing activity (DCL3 activity) was calculated as the relative band intensity of 24 nt RNA with respect to the intensity of all bands in each lane following autoradiography. Values are means ± standard deviation for three independent experiments. The schematic figure shows cleavage preference of DCL3. Solid arrows indicate cleavage preference of DCL3. Black stars indicate ^32^P.

To determine the preferred length of 3' overhang of substrate dsRNAs for DCL3 and DCL4, Dicer assays were performed using WT extract and 50 nt short dsRNA substrates with a blunt end, a 1 or 2 nt 3′ overhang structures (Figure 3A). DCL3 efficiently cleaved dsRNAs with a 1 or 2 nt 3′ overhang, whereas DCL4 cleaved all three types of dsRNAs with similar efficiency (Figure 3A). Product specificity for each DCL isoform was confirmed by assays using *dcl3-1* or *dcl4-2* extracts (Figure 3A). Both DCL3 and DCL4 are highly specific in cleaving the 5′ phosphorylated end of dsRNAs (black and gray arrowheads in Figure 3A). These results are consistent with the results shown in Figures 1 and 2. Products of cleavage by DCL4 from non-phosphorylated ends were detected (white arrowheads, Figure 3A) but not by DCL3 (Figure 3A, right panel), indicating that the recognition of 5′ phosphorylated nucleotides by DCL3 is more rigorous than that of DCL4.

When 37 nt dsRNAs with a 1–3 nt 3′ overhang or blunt end were used as substrates, DCL3 cleaved 37 nt dsRNAs with a 1 nt 3′ overhang more efficiently than those with a 2 nt 3′ overhang (Figure 3B). Taken together with the results of Figure 1, DCL3 preferentially cleaves short dsRNAs with a 1 nt 3′ overhang but DCL4 preferentially cleaves long dsRNAs.

### DCL3 produces a 24 nt RNA duplex with a 2 nt 3′ overhang by the 5′ counting rule

It is interesting that DCL3 preferentially cleaves short dsRNAs with a 1 nt 3′ overhang as well as those with a 2 nt 3′ overhang (Figure 3), because human Dicer preferentially cleaves dsRNAs with a 2 nt 3′ overhang into small RNA duplexes with a 2 nt 3′ overhang (16). Furthermore, human Dicer mainly determines the cleavage site by the distance from the 5′ end (the 5′ counting rule) (16) but Dicer from *Giardia intestinalis* measures the cleavage site from the 3′ end (the 3′ counting rule) (9). It will be important to determine whether the DCL3 cleavage products from dsRNAs with a 1 nt 3′ overhang have a 1 or 2 nt overhang at their 3′ end.

**Figure 4.**
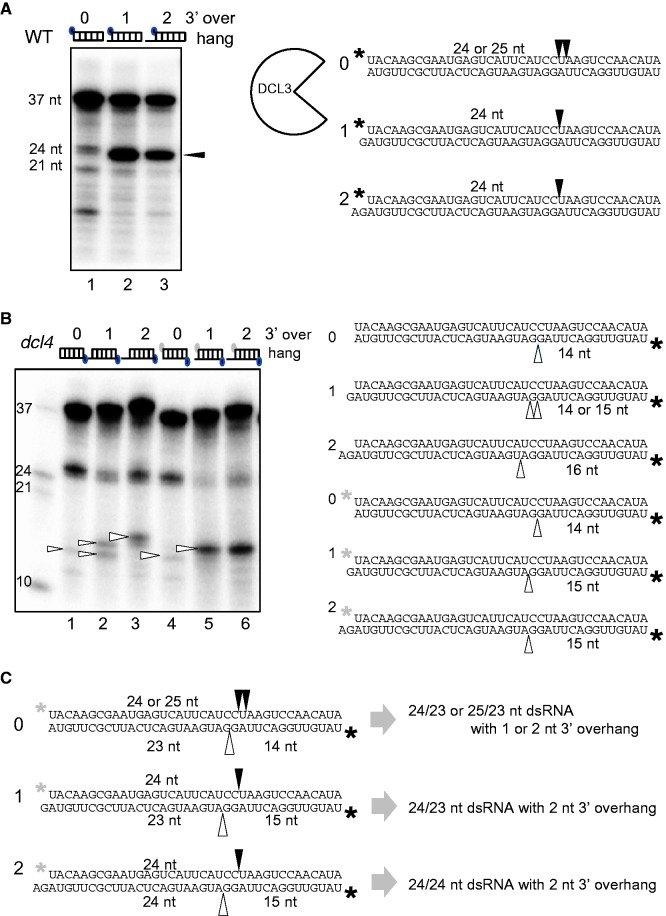
DCL3 produces 24 nt RNA duplex with 2 nt 3′ overhang by the 5′ counting rule. (**A**) Sites of cleavage by DCL3 from the 5′ phosphorylated end. Cleavage products formed from 37 nt dsRNAs with blunt ends (0) or 1 nt (1) or 2 nt (2) 3′ overhangs by DCL3 were analysed by denaturing PAGE. Black arrowheads indicate the cleavage products on PAGE and the cleavage sites on substrate dsRNA sequences. Black circles or stars indicate ^32^P at the 5′ phosphorylated end. (**B**) Sites of cleavage by DCL3 from the 3′ hydroxyl end. Dicer assays were performed using *dcl4-2* crude extracts. Cleavage products produced by DCL3 from two sets of 37 nt dsRNAs with blunt ends (0) or with 1 nt (1) or 2 nt (2) 3′ overhangs that carry either a phosphate or a hydroxyl group at the 5′ terminus were analysed. White arrowheads indicate the cleavage products on PAGE and the cleavage sites on substrate dsRNA sequences. Black circles (stars) and gray circles (stars) indicate ^32^P and cold 5′ phosphate (^31^P), respectively. (**C**) Structures of cleavage products produced by DCL3 are inferred. Black and white arrowheads indicate cleavage sites on each strand inferred by (A) and (B), respectively. Black and white stars indicate phosphates at 5′ ends.

We analysed the structure of products cleaved from 37 nt dsRNAs by DCL3. The cleavage sites are summarized in the right panel of Figure 4A, indicated by black arrowheads on the phosphorylated strand of substrate dsRNAs. In a Dicer assay with 37 nt dsRNAs with blunt ends, or with a 1 or 2 nt overhang at the 3′ end as substrates, most cleavage products from dsRNAs with either a 1 or 2 nt 3′ overhang were 24 nt (Figure 4A, lanes 2 and 3), suggesting that DCL3 accessed these dsRNAs from the 5′ phosphorylated end and cleaved these 5′ phosphorylated dsRNAs to produce small dsRNAs with a 24 nt 5′ phosphorylated strand (5′ counting rule; 16). However, DCL3 cleaved 37 nt dsRNA with blunt ends into 24 and 25 nt RNAs (Figure 4A, lane 1).

To determine the DCL3 cleavage site on the opposite strand of 37 nt dsRNAs (the lower strand of dsRNAs in a right panel of Figure 4B), the opposite 5′ termini of substrate dsRNAs were labeled by ^32^P. Two sets of 37 nt dsRNA substrates were prepared, carrying either a 5′-terminal phosphate or a hydroxyl group (Figure 4B) at the unlabeled termini. To measure DCL3 activity specifically, the assays were performed using crude extracts from *dcl4* seedlings.

The cleavage products of correct direction and their cleavage sites on the opposite strands of substrate dsRNAs are indicated by white arrowheads in Figure 4B. DCL3 cleavage products from the two substrate sets differed markedly. The cleavage sites from the phosphorylated ends of dsRNAs followed the 5′ counting rule, whereas those from the dsRNAs lacking the 5′ phosphate mainly obeyed the 3′ counting rule. This 5′ phosphorylation-dependent cleavage rule of DCL3 is similar to that of human Dicer (16).

The above results also reinforced the authenticity of DCL3 assay used in this study. Based on the length and structure of the DCL3 cleavage products (Figures 4A, lanes 1–3; and 4B, lanes 4–6), the major DCL3 cleavage products from 37 nt dsRNA substrates with a 1 or 2 nt 3′ overhang and 5′ phosphorylated termini were 24 nt dsRNAs with a 2 nt 3′ overhang (24 nt RNA duplex) (Figure 4C). This is consistent with the genome-wide profiling data of *Arabidopsis* siRNAs (32), indicating that our *in vitro* Dicer assay using crude extracts correctly reconstructed the *in vivo* DCL3 reaction. In addition, requirement of 5′ phosphorylated termini agrees well with the fact that small dsRNA produced *in vivo* by DCLs contain 5′ phosphorylated termini (11–13).

### Inorganic phosphate, NaCl and KCl enhance DCL3 activity but inhibit DCL4 activity

**Figure 5.**
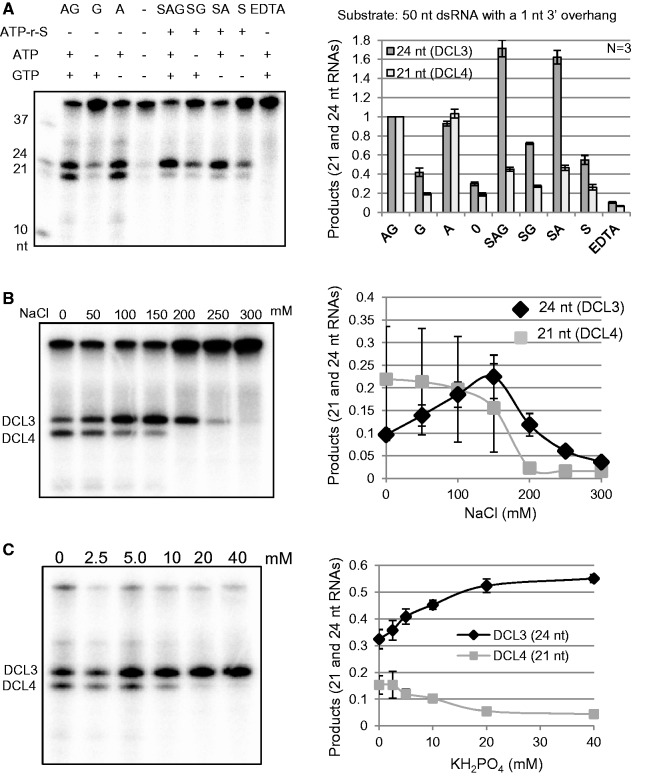
Inorganic phosphate, NaCl and KCl enhance DCL3 activity but inhibit DCL4 activity. (**A**) ATP and Mg ions are required for both DCL3 and DCL4 activities. Dicer assays for 50 nt dsRNA with a 1 nt 3′ overhang as a substrate were performed in reaction mixtures under various conditions. The standard Dicer buffer contained 30 mM Tris-HCl (pH 7.5), 50 mM NaCl, 4 mM MgCl_2_, 5 mM ATP and 1 mM GTP. To this buffer, 5 mM ATPγS or 4 mM EDTA was added. Sodium chloride (**B**) and inorganic phosphate (**C**) enhance DCL3 activity but inhibit DCL4 activity. Dicer assays were performed with various concentrations of NaCl (B) or KH_2_PO_4_ (C). Cleavage products from 50 nt dsRNA with a 2 nt 3′ overhang were analysed. The 21 nt RNA producing activity (DCL4 activity) was calculated as the relative band intensity of 21 nt RNA with respect to the intensity of all bands in each lane, and the 24 nt RNA producing activity (DCL3 activity) was calculated using the same equation. Values are means ± standard deviation for three independent experiments.

Various co-factors and ionic environments affect the dicing activities of DCLs (29–31). To determine if activity and substrate specificity of DCL3 and DCL4 could be regulated by cellular environment, we examined the dsRNA-cleaving activities of DCL3 and DCL4 under various conditions using 50 nt dsRNAs with blunt ends (Supplementary Figure S3A), 1 nt 3′ overhangs (Figure 5A), or 2 nt 3′ overhangs (Supplementary Figure S3B) as substrates. The analysis of dicing assay results indicated that both ATP and Mg^2+^ were necessary for the dsRNA-cleaving activities of both DCL3 and DCL4, but GTP was not essential for either activity (Figures 5, Supplementary Figure S3). In various conditions, DCL3 effectively cleaved dsRNAs with a 1 or 2 nt 3′ overhang (compare Figure 5A and Supplementary Figure S3B with Supplementary Figure S3A).

Adenosine 5′-O-(3-thio)triphosphate (ATPγS) is a non-hydrolysable ATP analog. Because ATP is required for both DCL3 and DCL4 activities, the effects of ATPγS on dsRNA cleavage by these proteins were examined (Figure 5A, Supplementary Figure S3A and B). ATPγS inhibited the dsRNA-cleaving activity of DCL4 but perhaps enhanced the dsRNA-cleaving activity of DCL3. When ATPγS was present in the reaction mixture, ATP was not necessary for DCL3 activity. Therefore, DCL4 may require ATPase activity for dsRNA-cleavage whereas DCL3 does not. ATP, but not ATP hydrolysis, is necessary for the dsRNA-cleaving activity of DCL3. DCL3 is different from DCL4 with respect to not only the substrate dsRNA preferences shown in Figures 1–3 but also the requirement for ATP hydrolysis for dsRNA cleavage.

We examined the dsRNA-cleaving activities of DCL3 and DCL4 in reaction mixtures with various concentrations of NaCl (Figure 5B and Supplementary Figure S4A) or KCl (Supplementary Figure S4B). The effect of NaCl and KCl on DCL activities was similar. The NaCl or KCl concentration affected the activity of both DCL3 and DCL4, but with marked differences. Both NaCl and KCl inhibited DCL4 activity but enhanced DCL3 activity up to ∼200 mM (Figure 5B and Supplementary Figure S4). NaCl at ∼150 mM or KCl at 150–200 mM was optimum concentrations for DCL3 activity but inhibited DCL4 activity (Figure 5B and Supplementary Figure S4). The optimum concentration of NaCl or KCl for DCL4 activity was 0–50 mM. It is surprising that the optimum NaCl or KCl concentrations for DCL3 and DCL4 differ markedly though both DCLs function in the cell nucleus (20). However, since the vast majority of plant viruses does not enter the nucleus (23), an antiviral Dicer, DCL4, likely functions in the cytoplasm, too.

Recently, it has been reported that inorganic phosphate inhibits pre-miRNA cleavage by *Drosophila* Dicer-2 (15). We examined whether inorganic phosphate affects dsRNA cleavage by DCL3 or DCL4. Interestingly, potassium phosphate (Figure 5C) or sodium phosphate (Supplementary Figure S5) at 10–20 mM, reported to be the physiological concentration of inorganic phosphate in plant cells (33), inhibited DCL4 activity but enhanced DCL3 activity. It was again surprising that inorganic phosphate differentially affected DCL3 and DCL4 activities. Inorganic phosphate may regulate the substrate specificities of DCL3 and/or DCL4, as it restricts the substrate specificity of *Drosophila* Dicer-2 (15).

The effects of temperature on DCL3 and DCL4 activities were examined. Using 50 nt dsRNAs with blunt ends or 1 or 2 nt 3′ overhangs as substrates, the activity of DCL3 toward these three dsRNAs increased as the temperature increased, but DCL4 activity did not change (Supplementary Figure S6). This temperature dependence of DCL3 and DCL4 activities may be consistent with the previous report that low temperature inhibits RNA silencing-mediated defence by the control of siRNA generation (34).

**Figure 6.**
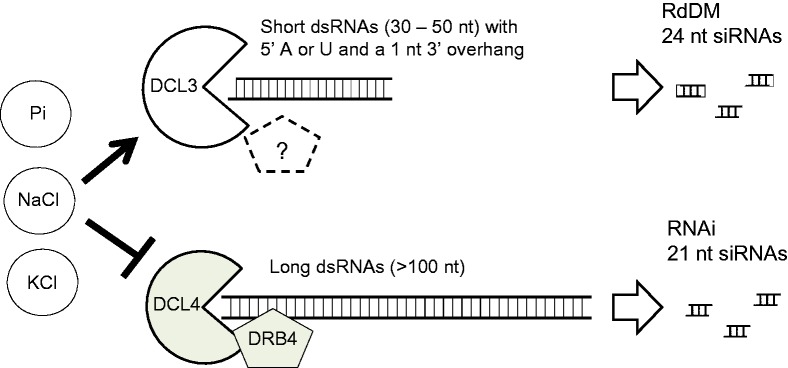
A graphical abstract of the distinct substrate specificities of *Arabidopsis* DCL3 and DCL4. DCL3 preferentially cleaves short dsRNAs of 30–50 nt with a 1 nt 3′ overhang and 5′ phosphorylated adenosine or uridine, whereas DCL4 preferentially cleaves dsRNAs of longer than 100 nt. Inorganic phosphate, NaCl and KCl enhance DCL3 activity but inhibit DCL4 activity.

Overall, the enzymatic properties of DCL3 and DCL4 differ markedly, several factors affecting their activities in the opposite directions (Figure 6). Furthermore, distinct substrate specificities of DCL3 and DCL4 are probably responsible for predisposing each dsRNA species to generate only specific size of siRNA *in vivo*.

## DISCUSSION

Plants have at least four Dicer proteins, and three of them are siRNA producing Dicers (DCL2 to 4) with distinct roles in each RNA silencing pathway (18,21). Concomitantly, the product of DCL3 and DCL4 (24 and 21 nt siRNAs, respectively), have distinct important functions *in vivo*; the longer siRNAs act in TGS via RdDM to suppress transposon activation and the shorter siRNAs act in PTGS via RNA cleavage to regulate gene expression and defend against viral infections. Although many reports genetically elucidated *in vivo* functions of DCL3 and DCL4 (20,22–24), substrate recognition mechanisms by these two Dicers have not been reported. Nonetheless, production of proper sized-siRNAs from proper dsRNA substrates generated from individual nucleic acid parasites by each Dicer protein must be crucial for protecting plants from transposon activation and viral infection.

In this study, we presented several important results for understanding the substrate recognition mechanisms of DCL3 and DCL4. In particular, the recognition of substrate dsRNA lengths by DCL3 and DCL4 (Figure 1) offers a reasonable explanation for the mechanisms generating siRNA from proper dsRNA substrates, because biological substrates for DCL3 may be short dsRNAs produced by RNA polymerase IV and RNA-dependent RNA polymerase 2, whereas biological substrates for DCL4 are long dsRNAs, such as ta-siRNA precursors, produced by RNA polymerase II and RNA-dependent RNA polymerase 6, or replication intermediates of viral genomic RNAs (20,22–24). Recently, it has been demonstrated *in vitro* that RNA polymerase IV produces shorter RNAs than RNA polymerase II does (35).

All data presented here were obtained using crude extracts from ∼2-week-old *Arabidopsis* seedlings. Because our Dicer assay is very simple, it is applicable to other *Arabidopsis* tissues and other plant species. Furthermore, crude extracts are likely to contain co-factors necessary for the dsRNA-cleaving reactions by DCL3 and/or DCL4. Reactions using crude extracts rather than recombinant proteins are likely more similar to *in vivo* conditions. Indeed, the structures of products cleaved from dsRNAs by crude extracts from the *dcl4* mutant (having only DCL3 activity) are consistent with the structure of 24 nt siRNAs found *in vivo* (Figure 4). Therefore, our Dicer assay may reconstitute *in vivo* reactions faithfully. On the other hand, since crude extracts contain various proteins, unknown co-factors that specifically associate with DCL3 or DCL4 might influence the substrate specificities of DCL3 and DCL4.

DCL3 recognizes the terminal structures of substrate dsRNAs, and the 5′ phosphate and 3′ overhang are important for recognition by DCL3. Recently, Park *et al.* (16) demonstrated that human Dicer recognizes the 5′ phosphorylated end of dsRNAs for efficient and accurate processing. This 5′ recognition model (5′ counting rule) is likely to be applicable to dsRNA cleavage by DCL3. DCL3 recognizes and accesses the 5′ phosphorylated end of short dsRNAs to efficiently and accurately process them into 24 nt RNA duplexes with a 2 nt 3′ overhang (Figures 3 and 4). In addition, DCL3 preferentially cleaves short dsRNAs with 5′ phosphorylated adenosine or uridine residues as substrates. Small RNA transcriptome analysis in *A. thaliana* indicated that adenine is the most common first base of the 24 nt small RNAs (36), and plant Argonaute proteins show distinct preferences of 5′ nucleotide in target siRNA (37,38). The cleavage preference of DCL3 for dsRNAs with 5′ adenosine may promote loading of resulting 24 nt product into specific Argonaute proteins (such as AGO4 and AGO6) to 24 nt small RNAs (39). At this point, we are not able to fully elucidate substrate specificities of DCL3 and DCL4 toward long dsRNAs with different overhangs, because only blunt end substrates were available (see MATERIALS AND METHODS section).

DCL4 is likely to be an ortholog of *Drosophila* Dicer-2, because both Dicers produce 21 nt siRNAs, which function in RNA interference (PTGS), from long perfect dsRNAs, and both are associated with partner dsRNA-binding proteins, R2D2 for Dicer-2 and DRB4 for DCL4 (7,31,40). Furthermore, their activities are dependent on ATP and inhibited by inorganic phosphate (15). *Drosophila* Dicer-2 requires ATP to processively cleave long perfect dsRNAs into 21 nt siRNAs but does not require ATP for a single cleavage of short dsRNAs or miRNA precursors (15). Cenik *et al.* (15) also proposed that the Dicer-2 helicase domain uses ATP to generate many siRNAs from a single long dsRNA. In this study, we demonstrated that DCL4 but not DCL3 preferentially cleave long dsRNAs (Figure 1), and requires ATPase activity for dsRNA cleavage (Figure 5). DCL4 may require ATP hydrolysis for cleaving long perfect dsRNAs efficiently (i.e., for multiple cleavages) whereas DCL3 does not require ATP for cleaving short dsRNAs with weak A-U base-pairing at the 5′ end (single cleavages).

The optimum NaCl or KCl concentration for DCL3 activity is 150–200 mM, which is unique because the dsRNA-cleaving activities of most Dicers reported so far, including human Dicer and *Arabidopsis* DCL1 and DCL4, are inhibited by 200 mM NaCl (Figure 5B) (6,30,31). No salt-activated Dicer has been reported previously. The biological significance of different optimum salt concentrations for two Dicers in the same plant species remains unknown.

Cenik *et al.* (15) reported that inorganic phosphate inhibits *Drosophila* Dicer-2′s dicing of pre-miRNAs but not its dicing of long perfect dsRNAs. Pre-miRNAs are not biological substrates for Dicer-2 but they are for Dicer-1. Cenik *et al.* concluded that inorganic phosphate inhibits Dicer-2 from cleaving pre-miRNAs, which are the wrong substrates. If this conclusion is applied to our results, inorganic phosphate inhibits cleavage of short (50 nt) dsRNAs by DCL4, which may not be its biological substrates (Figures 1 and 5). True biological substrates for DCL4 are likely to be dsRNAs of longer than 100 nt (Figure 1), whereas short dsRNAs are likely to be biological substrates for DCL3.

The amount of cleavage products produced from 50 nt dsRNAs by DCL3 and DCL4 was comparable in reaction mixtures containing no NaCl, KCl or inorganic phosphate (Figures 1–3 and 5), but the amount of 24 nt cleavage products formed by DCL3 was ∼10-fold greater than that of 21 nt products formed by DCL4 when the proteins were assayed in the presence of 200 mM NaCl or KCl or 20 mM inorganic phosphate (Figure 5B and C). These results suggest that the siRNA-generating activities of DCL3 and DCL4 may be regulated by cellular ion concentrations, which are influenced by environmental conditions.

## SUPPLEMENTARY DATA

Supplementary Data are available at NAR Online.

## FUNDING

A Grant-in-Aid for Scientific Research [No. 24570044]; a special research grant for advanced plant protection strategies from the Ministry of Education, Culture, Sports, Science and Technology of Japan (to T.F.); a collaborative research grant from the Collaborative Research Ring of Tokyo University of Agriculture and Technology (to T.F.). Funding for open access charge: a Grant-in-Aid for Scientific Research [No. 24570044]; Ministry of Education, Culture, Sports, Science and Technology of Japan.

*Conflict of interest statement.* None declared.
